# Comparability of multi‐temporal DTMs derived from different LiDAR platforms: Error sources and uncertainties in the application of geomorphic impact studies

**DOI:** 10.1002/esp.5540

**Published:** 2023-02-06

**Authors:** Nicole Kamp, Paul Krenn, Michael Avian, Oliver Sass

**Affiliations:** ^1^ Institute of Geography and Regional Science University of Graz Graz Austria; ^2^ FWF DK Climate Change University of Graz Graz Austria; ^3^ Department for Water Management Federal State Government of Carinthia Klagenfurt Austria; ^4^ Department of Earth Observation and Geoinformation Zentralanstalt für Meteorologie und Geodynamik Vienna Austria; ^5^ Chair of Geomorphology, Department of Geosciences University of Bayreuth Bayreuth Germany

**Keywords:** airborne LiDAR, digital terrain model, DTMs of difference, UAV‐borne LiDAR, uncertainty analysis

## Abstract

Multi‐temporal digital terrain models (DTMs) derived from airborne or uncrewed aerial vehicle (UAV)‐borne light detection and ranging (LiDAR) platforms are frequently used tools in geomorphic impact studies. Accurate estimation of mobilized sediments from multi‐temporal DTMs is indispensable for hazard assessment. To study volumetric changes in alpine environments it is crucial to identify and discuss different kind of error sources in multi‐temporal data. We subdivided errors into those caused by data acquisition, data processing, and spatial properties of the terrain. In terms of the quantification of surface changes, the propagation of errors can lead to high uncertainties.

Three alpine catchments with different LiDAR point clouds of different origins (airborne laser scanning [ALS], UAV‐borne laser scanning [ULS]), varying point densities, accuracies and qualities were analysed, and used as basis for interpolating DTMs. The workflow was developed in the Schöttlbach area in Styria and later applied to further catchments in Austria. The main aim of the presented work is a comprehensive DTM uncertainty analysis specially designed for geomorphic impact studies, with a resulting uncertainty analysis serving as input for a change detection tool. Our findings reveal that geomorphic impact studies need the careful distinction between actual surface changes and different data uncertainties. ULS combines the benefits of terrestrial laser scanning with all the benefits of ALS. However, the use of ULS data does not necessarily improve the results of the analysis since the high level of detail is not always helpful in geomorphic impact studies. In order to make the different point clouds and DTMs comparable the quality of the ULS point cloud had to be reduced to fit the accuracy of the reference data (older ALS point clouds). Using a point cloud with a high point density with a regular planimetric point spacing and less data gaps, in the best case collected during leaf‐off conditions (e.g., cross‐flight strategy) turned out to be sufficient for our geomorphic research purposes.

## Graphical abstract

Our work shows that geomorphic impact studies need a careful distinction between surface change and inherent data noise. A comprehensive DTM uncertainty analysis was applied to ensure the quality of geomorphic impact studies. ULS combines the benefits of TLS with all the benefits from ALS. Quality differences between ULS and ALS lead to significant limitations in the quality of geomorphic impact studies. The full potential of ULS data (high point density, representation of small‐scale structures) can only be used when being compared to data with a similar or same accuracy and quality.

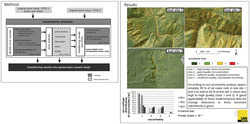

## 1 INTRODUCTION

Since the beginning of the 21st century, LiDAR (light detection and ranging; also known as laser scanning) has been a frequently used active remote sensing technique for studying geomorphic processes. LiDAR data increases the level of detail for geomorphological mapping and improves the quality of terrain data (Tarolli et al., 2009). The use of LiDAR data has increased exponentially as high‐resolution topographic data are nowadays widely available and accessible. Because of its ability to penetrate vegetation cover and acquire point data with high density, precision, and accuracy, LiDAR provides an accurate and high‐resolution representation of the earth's surface. New technological advances in geomorphology (Viles, 2016) and the transition from interpolating a digital terrain model (DTM) from a small amount of terrestrial surveyed terrain points to millions of LiDAR ground points improved the quality and reliability of geomorphometric analysis. Hence, high‐density LiDAR data has become a powerful asset in earth surface research (Fujii & Fukuchi, 2005; Jaboyedoff & Derron, 2020; Shan & Toth, 2017; Vosselman & Maas, 2010). Several laser scanning systems exist such as terrestrial laser scanning (TLS), airborne laser scanning (ALS), and mobile laser scanning (MLS) systems (e.g., mounted on a car or boat). A rather recent development in LiDAR technology is the application of MLS‐systems mounted on light‐weight uncrewed aerial vehicles (UAVs; UAV‐borne laser scanning [ULS]) for monitoring comparably small study areas (< 5 km^2^) (Bremer et al., 2019a). The significant advantage of ULS is that the benefits of TLS (e.g., short range or different scan angle) are combined with all the benefits from ALS (Mandlburger et al., 2015a).

While there are numerous projects of studying geomorphic impacts with the help of multiple terrestrial and airborne LiDAR data (Avian et al., 2018, 2020; Eitel et al., 2016; Goodwin et al., 2017; Jones et al., 2015; Niculiță et al., 2020; Schaffrath et al., 2015; Victoriano et al., 2018), there are only a few applications with ULS data since its development has only started over the last few years. The ability of ULS to collect very high point density data makes this new technique particularly popular in the field of vegetation and forest science (Hyyppä et al., 2020; Ivushkin et al., 2019; Mandlburger et al., 2015c; Risbøl & Gustavsen, 2018; Wallace et al., 2012; Węgrzyn et al., 2020). In geomorphologic context ULS is for example used to monitor erosion in alpine grassland (Mayr et al., 2019) or to study mass movements (Bremer et al., 2019a, 2019b; Eker et al., 2017; Zieher et al., 2019).

Both ALS and ULS systems use a laser scanner combined with a Global Navigation Satellite System (GNSS) and an inertial measurement unit (IMU). A LiDAR sensor transmits a light pulse towards the surface and measures the time the pulse takes to the surface and back. The sum of reflected pulses forms a three‐dimensional (3D) point cloud. The spatial location of the individual points is determined by the position of the sensor platform, the propagation time, and the angular measurements of the laser pulse to the surface (Fujii & Fukuchi, 2005; Shan & Toth, 2017; Vosselman & Maas, 2010).

The actual measurement principle of both systems is congruent, but due to shorter sensor‐object distances, the resulting point clouds of ULS provide higher point densities. Different scanning geometries (e.g., larger angle of incidence) caused by the lower/different flight height of the UAV also lead to smaller uncertainties and a more precise representation of the surface (Bakuła et al., 2017; Bakuła et al., 2020; Davidson et al., 2019; Mandlburger et al., 2015a; Pilarska et al., 2016). ULS sensor systems perform at higher accuracy, precision, laser pulse repetition rate and scan angle ranges (330° vs. 45°/60° in ALS). Compared to ALS, the processing of ULS point clouds is more complex due to higher point density, small footprints caused by the lower flight height and, resulting from this, the higher level of detail of geomorphic structures.

### 1.1 Error sources and uncertainties in LiDAR data

Geomorphic impact studies and the calculation of volumetric changes need a rigorous analysis of error sources and uncertainties. This is crucial to avoid a propagation of errors and to distinguish real geomorphic changes from changes due to different errors in the data to derive a reliable change detection (Anderson, 2019; Bangen et al., 2014; Brasington et al., 2000; Cavalli et al., 2008; Lane et al., 2003; Wheaton et al., 2010).

Our work aims to identify and quantify errors and uncertainties and subsequently discuss the comparability of multi‐temporal LiDAR data (point clouds and calculated DTMs) of different origin by evolving the approach of Wheaton et al. (2010). Wheaton et al. (2010) presented two robust methods on a cell‐by‐cell basis to estimate uncertainties in DTMs (respectively digital elevation models [DEMs]) and in a further step apply these uncertainties to geomorphic change detection by applying a lower threshold to the amount of surface change. The first approach uses a fuzzy inference system to calculate the spatial variability of uncertainties in multi‐temporal DTMs; the second approach modifies this estimation by discriminating uncertainties in DTMs of difference (DoD) on the basis of the spatial coherence of erosion and deposition units. Another advanced approach to estimate uncertainty by subdividing uncorrelated, correlated, and systematic errors was proposed by Anderson (2019). However, for the catchment‐wide scale of our work we decided to evolve the approach of Wheaton et al. (2010).

In our study, we compared and analysed ULS and ALS data of different point cloud densities derived with different LiDAR sensors in our study area Schöttlbach valley, a torrential catchment in Styria (Austria). Two additional evaluation areas were used to verify and improve our uncertainty analysis. The quality of LiDAR‐point clouds, processed DTMs, and their uncertainties are mainly influenced by the acquisition method, the processing method of the point clouds and DTMs and the spatial surface properties of the terrain. A short summary of these error sources in LiDAR data is given in Table 1.

**TABLE 1:** Summary of the error sources in LiDAR (light detection and ranging) data

Type	Short description	References
*Data acquisition*		
Instrumental errors	Technical limitations and inherent bias of the scanner	Anderson, 2019; Avian et al., 2018; Chaplot et al., 2006; Mandlburger et al., 2009; Wasklewicz et al., 2013; Wise, 2000
	errors caused by the aircraft (e.g., transmitted vibrations from the aircraft)	
	physical causes (temperature, air quality, clouds, moisture, etc.)	
Methodical errors	Flight planning errors (adjustment of flight height, flight speed, scan angle, operation time, and flight direction, strip overlapping, point density, data gaps, footprint, etc.)	
	operator errors	
Random errors	Random noise	
*Data processing*		
Errors in point clouds	Errors in point cloud pre‐processing (absolute and relative orientation, incorrect geo‐referencing, inadequate strip adjustment, etc.)	Aguilar et al., 2005; Avian et al., 2018; Bater & Coops, 2009; Davidson et al., 2019; Heritage et al., 2009; Polat & Uysal, 2015; Pfeifer & Mandlburger, 2017; Sithole & Vosselman, 2004; Wise, 2000
	filtering errors	
	classification errors ([mis‐]classification of points in ground and non‐ground points)	
Errors in processed digital terrain models (DTMs)	DTM interpolation errors (inappropriate interpolation algorithm or spatial resolution)	
*Spatial properties of the terrain*		
Surface errors	Factors influencing the vertical and horizontal accuracy of a DTM and spatial pattern of errors (slope characteristics, surface roughness, curvature, micro relief structures, size of surveyed structure	Avian et al., 2018; Chaplot et al., 2006; Fisher & Tate, 2006; Hyyppä et al.*,* 2005; Lallias et al., 2014; Stereńczak et al., 2016
	errors caused by dense vegetation or forest cover	
Physical errors	Reflectivity of a surface	
	albedo	

Data acquisition errors can be divided into instrumental, methodical errors and random errors. Instrumental errors are caused by the sensor system and by the used aircraft. The laser scanning system is limited by its internal accuracy and precision (Table 2) as the inherent system bias can lead to distortions in all three dimensions. Further errors can be caused by temperature, air quality and moisture (Wise, 2000). Transmitted vibrations from the aircraft are responsible for artefacts and errors in the final point clouds. Errors in flight planning, like the adjustment of flight height, scan angle, operation time, and flight direction, strip overlapping, point density, data gaps and operator errors are summarized to methodical errors. While instrumental and random errors should not be neglected, they are only marginally addressed in this work. Random errors are random noise and have to be filtered out of the point cloud. Instrumental and methodical errors are linked to topographic complexity (Chaplot et al., 2006) and are therefore reflected in the results of our uncertainty analysis.

**TABLE 2:** Technical specifications of the two LiDAR (light detection and ranging) systems and project‐specific parameters of the study area and the two evaluation areas described in Section 4.3. (Bremer et al., 2019a; Riegl Laser Measurement Systems, 2021; Vermessung AVT‐ZT‐GmbH, 2015; Vermessung AVT‐ZT‐GmbH, 2019, 2020)

	Airborne specification 1	UAV‐borne specification	Airborne specification 2
Point cloud code	Schöttlbach‐ALS^a^; Lorenzerbach‐ALS1^a^; Lorenzerbach‐ALS2; Rettenbach‐ALS1^a^	Schöttlbach‐ULS	Rettenbach‐ALS2
*System‐specific parameters*			
Sensor system	Riegl LMS‐Q560/LMS‐Q680i^b^	Riegl VUX‐1LR	Riegl VQ‐780i
Aircraft type	helicopter	UAV	airplane
System weight (kg)	20/17.5	3.75	20
Accuracy/precision of the scanner (mm)	20/20	15/10	20/20
Total laser pulse repetition rate	100/400 kHz	820 Khz	1 MHz
Maximum measurement range (m)	1.500/3.000	1.540	6.800
Scan angle range (deg)	45/60	330	60
Laser beam divergence (mrad)	≤ 0.5	≤ 0.5	≤ 0.18/ ≤ 0.25
Total measurement rate (meas/s)	240.000/300.000	750.000	666.000
Wavelength (nm)	1.550 (NIR)	1.550 (NIR)	1.064 (NIR)
*Project‐specific parameters*			
Average flight height (m)	~ 130–1300	~ 90	~ 1000–1100
Geo‐referencing	Surveyed fitting surfaces	RTK without GCP – fitting surfaces from ALS dataset	Surveyed fitting surfaces
Vertical/horizontal accuracy (cm)	±15/40	± 7/7	± 3/6
Strip overlap (%)	40–50	Single, not overlapping strips	50 (cross‐flight; 500 m strip spacing)
Projection/height system	ETRS89 – UTM‐33 N (EPSG: 25833) /levelled heights (Triest 1875, EPSG: 5778)	ETRS89 – UTM‐33 N (EPSG: 25833) /levelled heights (Triest 1875, EPSG: 5778)	ETRS89 – UTM‐33 N (EPSG: 25833) /levelled heights (Triest 1875, EPSG: 5778)
Flight date	2009–2012	November 2018	December 2018
Customer	Styrian Government/WLV	University of Graz	City surveyor's office of Graz

^a^ Data from first flight campaigns were used as reference data.

^b^ Survey was done with a double scanner.

Errors in the processing procedure are divided into point cloud and DTM‐based errors. Examples of point cloud errors are incorrect geo‐referencing of the point cloud, inadequate strip adjustment, incorrect or poor filtering, random noise, and (mis‐)classification of points in ground and non‐ground points (Aguilar et al., 2005; Bater & Coops, 2009; Pfeifer & Mandlburger, 2017; Polat & Uysal, 2015; Sithole & Vosselman, 2004).

Spatial properties of the terrain are split into surface and physical errors.

Terrain slope, roughness, curvature, low vegetation and forest cover in connection with operation time and flight height as well as the reflectivity of a surface or surface characteristics like object size, albedo, and reflectivity are the most important factors influencing the vertical and horizontal accuracy of a DTM and spatial pattern of errors (Avianet al., 2018; Chaplot et al., 2006; Hyyppä et al., 2005; Mandlburger et al., 2009, 2015b, 2015c; Stereńczak et al., 2016). Slope characteristics or micro‐relief structures characterize the terrain and affect data acquisition as well as data processing. These measured points in rough terrain caused by small‐scale structures can be spatially unrepresentative and lead to errors in final models. Steep slopes, however, are causing irregularities in signal reflection. Signal attenuation and fallout are forced by varying reflectivity (wavelength of the sensor system) of different objects, land‐cover types and surfaces (Fisher & Tate, 2006). For example, data collected from scanners which broadcast in the near‐infrared (NIR)‐range may have data gaps or low ground point densities over water, snow and ice‐bodies, in addition to areas with a dense vegetation cover (Lallias‐Tacon et al., 2014).

Based on the detailed analysis of different error sources, a comprehensive DTM uncertainty workflow is presented in our article. We introduce a workflow for assessing and evaluating quality, uncertainties, and comparability of DTMs used for geomorphic impact studies, identifying parameters and requirements which significantly affect the quality and the comparability of DTMs. Furthermore, we discuss data acquisition and processing errors of our data pool. The main uncertainty analysis is based on data on sediment mobilization in a highly active torrential catchment (Schöttlbach creek, Styria, Austria). The detailed outcome of this study in terms of sediment budgeting is presented in a second article in the same volume (Krenn et al., in review). The developed workflow was applied to data from two further catchments in Styria. The link between the examples presented is that in all cases, DoD were derived from datasets of different origins and densities.

The overarching research questions for our study are:

- How can errors and uncertainties in calculated DTMs be assessed and quantified to serve as a basis for accurate and more reliable geomorphic impact studies?
- Which uncertainties and challenges arise from comparing ALS and ULS datasets, exemplified by our case study in the catchment area Schöttlbach?
- What are the advantages and disadvantages of using UAV‐borne LIDAR point clouds in geomorphic impact studies?

## 2 STUDY AREAS

In this article, we subdivided our study areas in a training area (Schöttlbach catchment) in which the workflow for data evaluation was developed, and two evaluation areas (Lorenzerbach and Rettenbach catchments). The detailed geomorphological analysis of the Schöttlbach data is presented in a second article (Krenn et al., in review).

### 2.1 Training area

**FIGURE 1:**
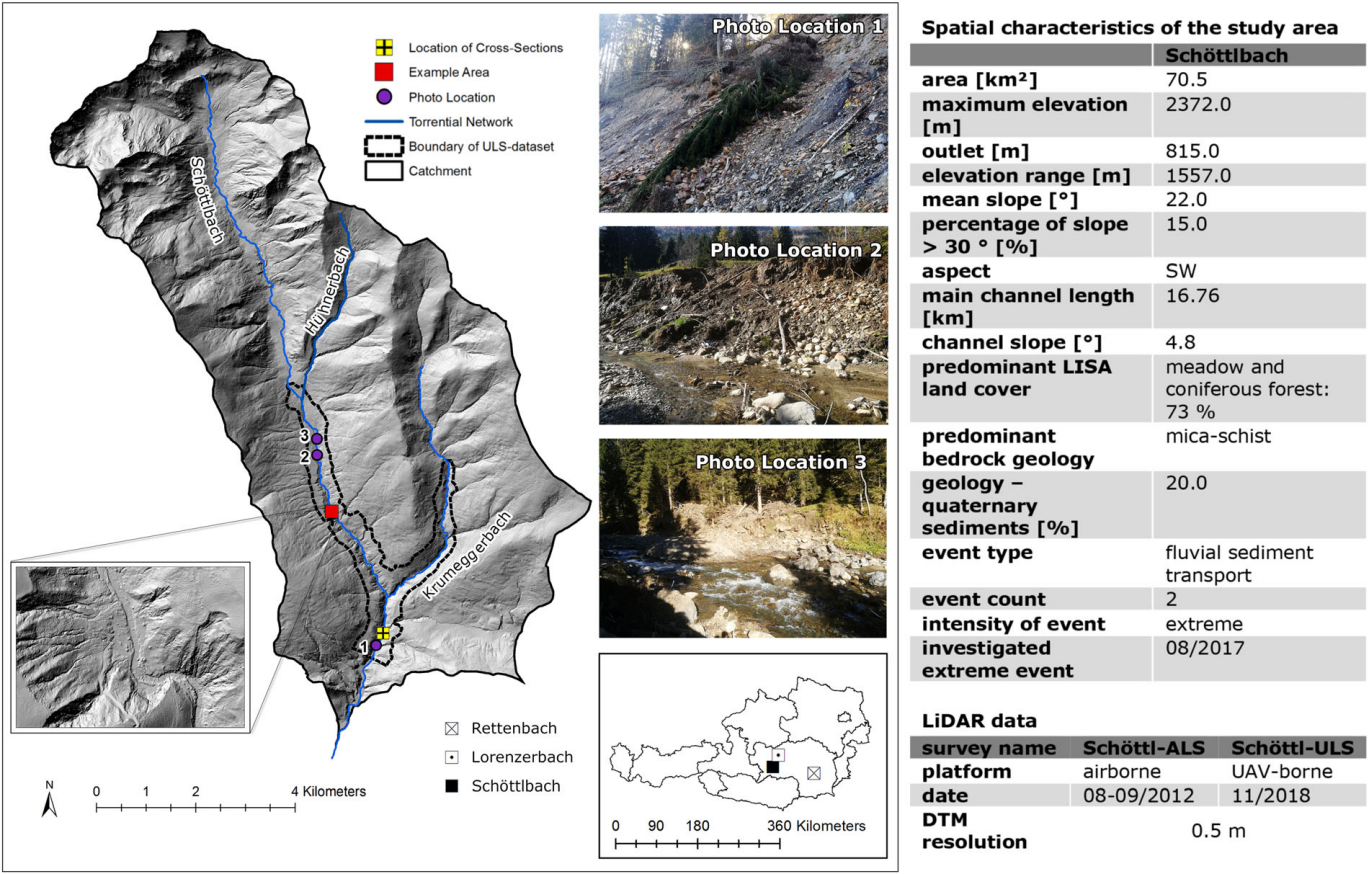
Location and basic information about the spatial characteristics of the Schöttlbach study area (hillshade image with 1.0 m spatial resolution, GIS Steiermark, 2021; images by Stefan Gsell, 2017). Photograph locations are indicated by purple points, locations of the later discussed cross‐sections by yellow squares with a black cross. The example area used for discussing the results is indicated by a red square. [Colour figure can be viewed at wileyonlinelibrary.com]

The Schöttlbach catchment is located near the small town of Oberwölz in Upper Styria (Austria) in the Niedere Tauern mountain range, which is part of the Austrian Central Alps (Figure 1). The catchment is 70.5 km^2^ wide; the bedrock consists of mica‐schist (75% of the area). The catchment is a highly active alpine torrential valley. It is dominated by meadows and coniferous forests (73% of the whole catchment) which partly reduce the number of reliable ground surface points in the LiDAR data. The valley opens to the southwest and is characterized by a considerable portion of steep slopes (15% steeper than 30°), particularly in the vicinity of the creek. The area was affected by heavy precipitation in August 2017, triggering a torrential flood event which mobilized estimated 130,000 m^3^ of sediments and caused considerable damage in Oberwölz. LiDAR scans were performed in late summer 2012 (Schöttlbach‐ALS) and November 2018 (Schöttlbach‐ULS). We used these datasets to develop a workflow for volume calculation and uncertainty assessment. The hydrological and geomorphological impacts of the event are reported in a second article (Krenn et al., in review).

The Schöttlbach catchment was used as training area to develop our uncertainty workflow.

### 2.2 Evaluation areas

**FIGURE 2:**
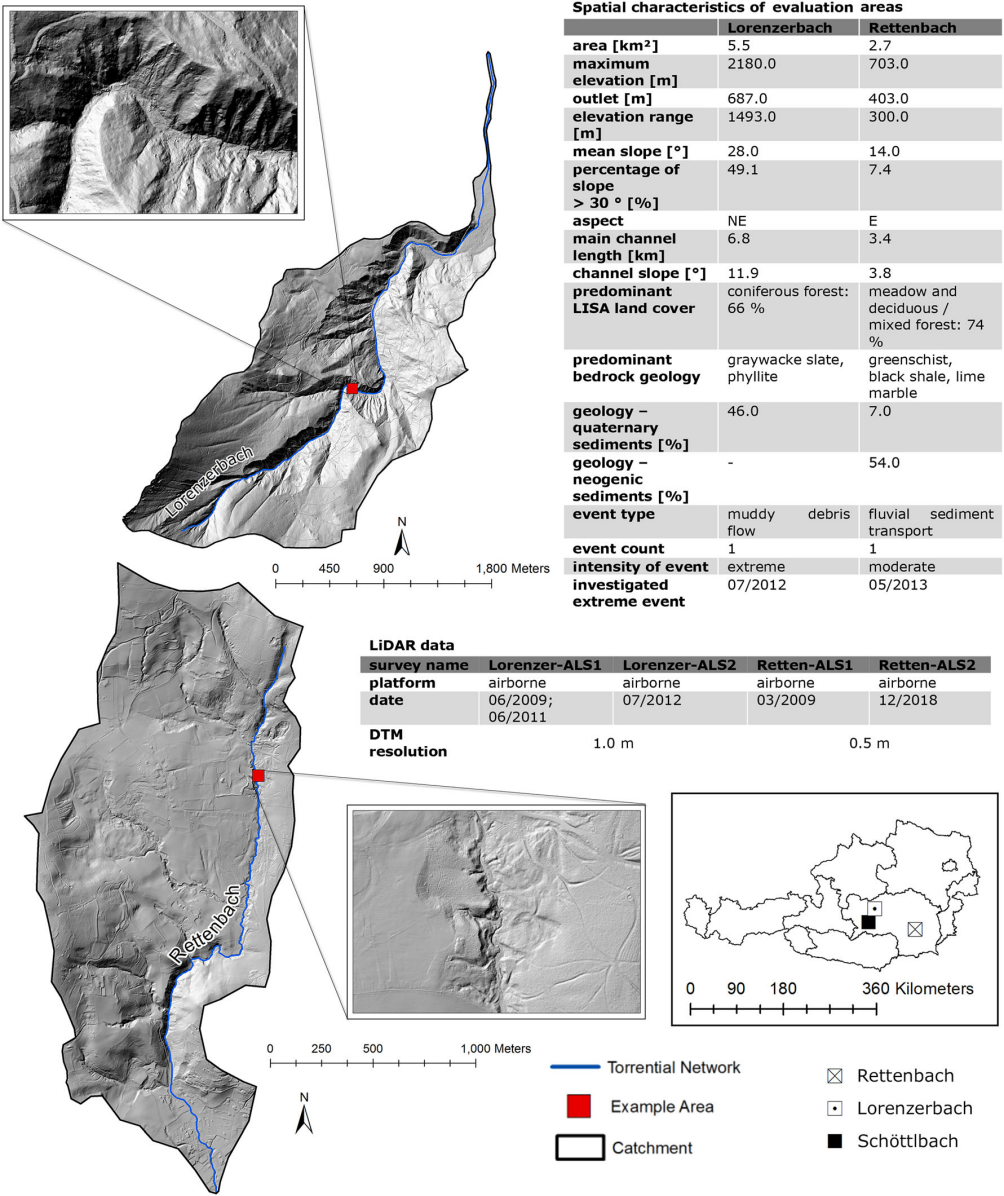
Location and spatial characteristics of the evaluation areas Lorenzerbach and Rettenbach (hillshade image with 1.0 m spatial resolution, GIS Steiermark, 2021) as well as technical specifications of LiDAR data. Example areas for discussing results are indicated by red squares. [Colour figure can be viewed at wileyonlinelibrary.com]

In order to assess the performance of the workflow and its applicability for geomorphic impact studies, additional datasets from other alpine catchments with recent geomorphic activity were used (Figure 2). The Lorenzerbach valley is also located in the Niedere Tauern range, while the Rettenbach valley is located in the Grazer Bergland (Figure 2). Both evaluation areas were also affected by damaging torrential events (Lorenzerbach: July 2012; Rettenbach: May 2013). Additional details about spatial characteristics were derived from geographic information system (GIS)‐based terrain analysis (Figure 2), key information of the different LiDAR surveys is summarized in Table 2.

## 3 MATERIALS AND METHODS

In the Schöttlbach area, the surface of the lower part of the Schöttlbach and the Krumegger channel was surveyed by ULS in November 2018 (Figure 1) in order to quantify sediment relocation caused by the extreme event of August 2017 (Krenn et al., in review). The ALS‐derived point cloud of autumn 2012 was used as reference. Change detection analysis was carried out using the QuantumGIS (QGIS) 3.16.1 Change Detection Tool (QCD‐tool) developed by the first author for the presented study (Krenn et al., in review). This open‐source python‐based change detection tool, which is part of the pyAlpineRisk toolbox (available via GitHub), is used to detect, calculate, and visualize surface and volume changes using two discrete DTMs. It was designed to identify errors and quantify uncertainties in multi‐temporal LiDAR data of different origin, thus enabling accurate change detection and reliable volume calculations from generated DTMs as well as point clouds. For this purpose, results from our DTM uncertainty analysis described in this article can be included in the QCD‐tool.

### 3.1 Data acquisition

**FIGURE A2:**
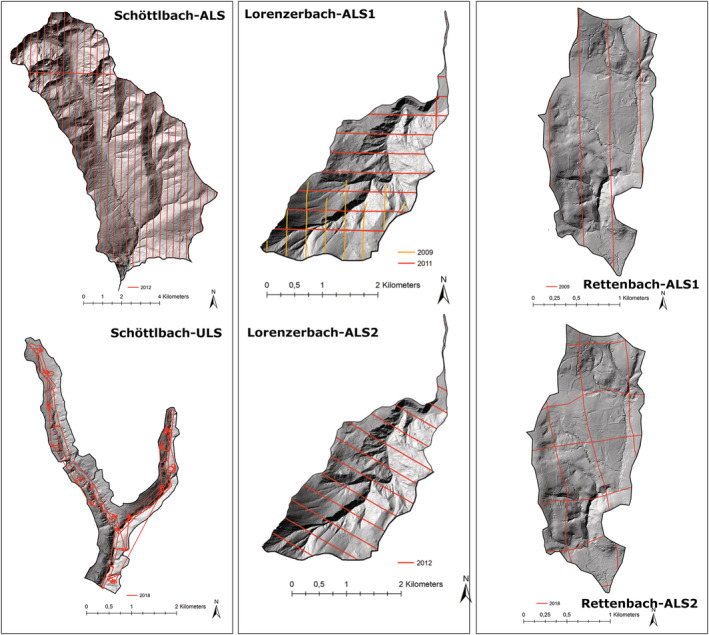
Flight paths of the different LiDAR point clouds. Different variants flight paths can be seen in the study and evaluation areas. [Colour figure can be viewed at wileyonlinelibrary.com]

The reference point cloud at Schöttlbach (training area) in 2012 was obtained by the federal‐state‐wide flight campaign (2008–2014), using an ALS double scanning system (RIEGL LMS‐Q560 and LMS_Q680i) mounted on a helicopter. For the ULS campaign in 2018, a UAV equipped with the lightweight RIEGL VUX‐1LR sensor (University of Innsbruck, Austria) was used (Table 2). Due to the elongated shape of the study area the flight plan for the ULS campaign followed two longitudinal lines along the river course. For detailed information see Appendix, Figure A2.

All point clouds were collected during dry, snow‐ and ice‐free weather conditions.

For the evaluation areas, the older ALS point clouds were also used as reference dataset (Figure 2 and Table 2, airborne specifications 1 and 2). The reference point cloud of Lorenzerbach from June 2009 did not cover the whole area and was therefore merged with a second point cloud from June 2011. The second point cloud in this area was acquired with the same technical setup as the reference point cloud. At Rettenbach, another ALS sensor (Table 2) was utilized for the second campaign in December 2018. This survey was carried out by using a cross‐flight strategy (with 500 m strip spacing) and with another sensor system (Table 2). Additionally, data were georeferenced with a high vertical (root mean square error [RMSE]: ±3 cm) and horizontal accuracy (RMSE: ± 6 cm). All point clouds were collected during dry, snow‐ and ice‐free weather conditions.

### 3.2 Data processing

The main data processing (georeferencing, strip adjustment, filtering, classification, etc.) was done by the respective operating LiDAR companies (ALS point clouds: Vermessung AVT‐ZT‐GmbH and ULS point cloud: Department of Geography, University of Innsbruck). A precise compliance of the quality parameters (like strip adjustment, mean ground point density or classification) of the different LiDAR campaigns was verified by additional commissioned LiDAR companies.

Based on this basic pre‐processing, an additional quality check was carried out by us to prepare data for further analysis and ensure data comparability.

The ULS point cloud was referenced at University of Innsbruck by real‐time kinematic (RTK) positioning without using ground control points (GCP). Stable surfaces (e.g., roof areas) within the ALS point cloud were used for matching the ULS to the ALS point cloud. This means that we used the Schöttlbach‐ALS as reference data for further processing (additional height adjustment) and quality control of the ULS point cloud. We used stable surfaces with different orientations and evenly distributed over the study area to verify the relative vertical and horizontal accuracy of the ULS point cloud. For this purpose, we applied the software package LAStools for classification, filtering, height above ground adjustment of the raw point clouds and interpolation of the respective DTMs from the ground points. LAStools (rapidlasso GmbH, 2021) can process large datasets with minimal computer processing power and in a short amount of time.

Since we used LAStools for data processing, we had to use either a TIN approach with standard linear interpolation (las2dem/blast2dem) or a grid‐based approach by computing the highest, lowest or average‐*z*‐value of all ground points within a cell (lasgrid). We used the TIN approach according to Fuller and Hutchinson (2007) who used this approach for fluvial environments. The spatial resolution of the raster had to be approximated to the underlying continuous terrain as well as the point density to reduce errors in the final DoDs (Fisher & Tate, 2006). Based on the different point densities of the data and evaluating QCD‐results determined with different spatial resolutions, a 0.5 m spatial resolution provided the best results for the Schöttlbach DTMs.

The software was originally designed for ALS point cloud processing; the ULS dataset required more manual post‐processing (e.g., manual filtering and classification) to guarantee the best possible results. Therefore, a manual re‐classification of the ULS ground points focusing on the small‐scale structures along and in the riverbed was required. The quality of the DTM and in a further step any kind of hydro‐morphological analysis would be substantially affected if misclassified objects were not corrected (e.g., boulders classified as vegetation).

### 3.3 Spatial properties of the terrain

Aguilar et al. (2005) and Heritage et al. (2009) emphasize that the quality of a DTM is influenced by the interpolation method, the ground point density, as well as the morphology of the terrain. More precisely, different factors are influencing the vertical and horizontal accuracy of a DTM and spatial pattern of errors (Hyyppä et al., 2005; Stereńczak et al., 2016) such as geomorphometry (slope gradient, micro‐relief), land‐use/land‐cover characteristics (reflectivity of a surface, low vegetation, forest cover) and measurement conditions (operation time, flight height).

Points in rough terrain can be spatially unrepresentative due to small‐scale structures and lead to errors in the final DoDs. Signal attenuation and point failures are affected by varying reflectivity of different slope gradients and objects, land‐cover types and surfaces, refraction, or light absorption. Water bodies (e.g., the water‐filled riverbed) may cause signal attenuation or even complete point failures while using a NIR sensor system (Fisher & Tate, 2006; Lallias‐Tacon et al., 2014).

The surface roughness $\sigma_{\text{rough}}$ of landforms and that is of natural targets (geomorphic structures) is the most important terrain parameter influencing the absolute DTM height accuracy (Mandlburger et al., 2015b).

The spatial geomorphometric properties of the terrain (mean slope, aspect, terrain roughness, elevation range, etc.) were calculated by applying a GIS‐based terrain analysis (Figures 1 and 8). The official torrent network, provided by the Austrian Service for Torrent and Avalanche Control (WLV), was used to derive main channel length and mean channel slope. Land cover and geology were taken from the Austrian land cover map LISA (Land Information System Austria, 10 m spatial resolution) and the geological map of Styria, scale of 1:50,000 (Geological Survey of Austria; adapted by Joanneum Research). Results were obtained by using the open‐source software products QGIS 3.16.1, SAGA GIS 2.3.2 and Python 3 (modules: gdal & numpy).

### 3.4 Analysis of error sources and uncertainties in multi‐temporal data

Due to the complex morphology in the study areas and heterogeneous quality and accuracy of the available point clouds and DTMs, a robust approach for the assessment of the comparability of multi‐temporal DTMs is needed. In the case of geomorphic impact studies, it is necessary to distinguish between actual surface changes and an inherent noise in the datasets in order to calculate accurate erosion and deposition volumes.

**FIGURE 3:**
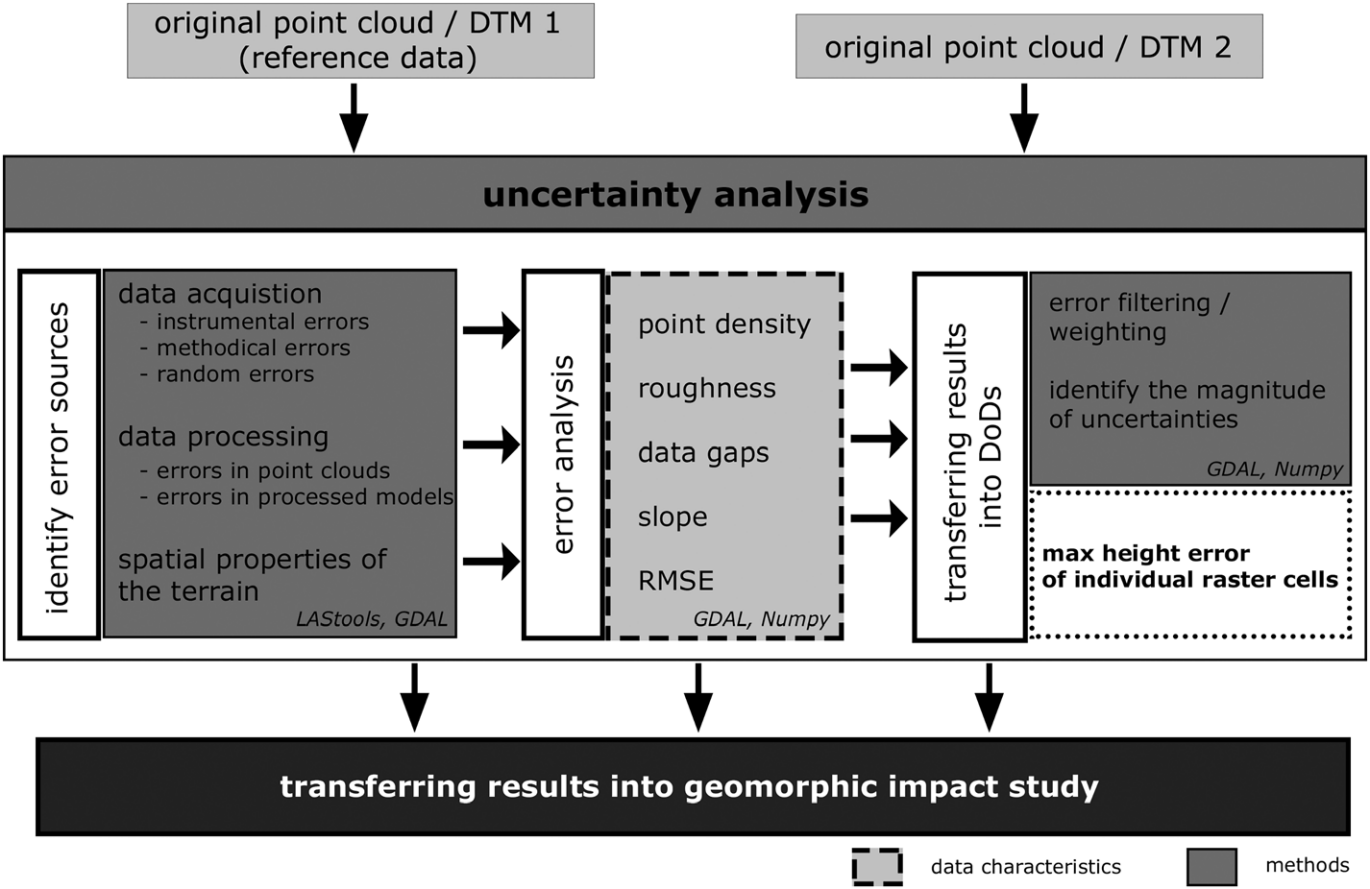
Uncertainty analysis in multi‐temporal data: schematic workflow of the uncertainty analysis used for geomorphic impact studies

Based on the approaches of Voltz and Webster (1990), Kraus et al. (2006), and Wheaton et al. (2010), we developed a workflow for identifying and assessing errors and uncertainties in DTMs (Figure 3). Three essential steps were taken for a robust error and uncertainty analysis in DTMs and DoD:

- Detecting possible sources of error in the different datasets,
- transferring these results into the DoDs,
- estimating model uncertainties by calculating cell by cell deviations.

The results of the uncertainty analysis gathered during this process was transferred into the geomorphic impact study to estimate upper and lower thresholds of the sediment budget (Krenn et al., in review).

As recommended by Reuter et al. (2009) and Wasklewicz et al. (2013), we analysed all different error sources described in Section 1 (Table 1) with the help of visual analysis, evaluation of the information provided by the operating LiDAR companies and LAStools. We tried to keep errors as low as possible with regard to data processing and quantified all remaining uncertainties by using a GIS‐supported statistical‐empirical modelling approach on a cell‐by‐cell basis described in this section. This analysis was done in two main steps: Step 1 – DTM height accuracy estimation; Step 2 – uncertainty analysis.

#### 3.4.1 DTM height accuracy analysis

For a realistic DTM height accuracy estimation, the calculation of the accuracy in height of each interpolated raster cell is essential (Kraus et al., 2006). For our study, we assumed that the relative accuracy of the interpolated points is (1) influenced by point density and point spacing of the underlying original point cloud; (2) the absolute accuracy of the original points; (3) different terrain characteristics like steep terrain, micro‐relief structures, or terrain roughness (Hyyppä et al., 2005; Kraus et al., 2006; Stereńczak et al., 2016). In terms of terrain characteristics, it is assumed that DTM height accuracy errors are higher, the more complex terrain structures are (Liu & Jezek, 1999). We presumed that the georeferencing of the different point clouds was carried out carefully with a high accuracy and therefore ignored the factor ‘absolute accuracy’ in this study. Unlike Voltz and Webster (1990), who used a sample of validation points to verify the DTM height accuracy, we used the original LiDAR point clouds ( $Z_{\text{PointCloud}}$) as reference, instead of collecting GCPs, and compared it with the modelled *z*‐value for the interpolated surface (Bater & Coops, 2009). The interpolated $Z_{\mathrm{DTM}}$ values were subsequently compared with the original point cloud $Z_{\text{PointCloud}}$ (| $Z_{\text{PointCloud}}$– $Z_{\mathrm{DTM}}$|) to calculate the deviations of original point data from the gridded and simplified DTM. This is in contrast to Wheaton (2008), who applied a bootstrapping approach (removing random samples of 10% of the points, while calculating several DTMs) to measure the quality of the interpolated elevation values.

This estimation was done for both DTMs. The result was a DTM height accuracy for each raster cell with the same resolution than the analysed DTM. Each cell provides information of the maximum deviation of original point data from $Z_{\mathrm{DTM}}$. In a next step, the determined DTM height accuracy was classified into three classes:

- good DTM height accuracy (low surface roughness): mean height deviations of < 0.1 m
- medium DTM height accuracy (medium surface roughness): mean height deviations of 0.1 to 0.2 m
- low DTM height accuracy (high surface roughness): mean deviations > 0.2 m

The classification, which was needed for the uncertainty analysis, is based on the precision/accuracy of the scanner and a detailed evaluation of the DTM height accuracy models (Table 2).

Our approach also provides accurate information on surface roughness $\sigma_{\text{rough}}$(Bater & Coops, 2009), which also depends on the point density of the original point cloud, the underlying terrain and the spatial resolution of the DTM (Bater & Coops, 2009; Mandlburger et al., 2015b). In addition, our approach also shows how well the individual flight strips are adjusted to each other.

#### 3.4.2 Uncertainty analysis

The values of uncertainty of each single raster cell within DoDs were analysed, combined, and classified making use of the following information: (1) RMSE of the discrepancies in height of the used DTMs, (2) point density, (3) slope, (4) data gaps, and (5) height accuracy of the multi‐temporal DTMs as well as of the point clouds (Figure 3).

1. We used the RMSE ( $Z_{{\mathrm{DTM}}_{\mathrm{old}}},\;Z_{{\mathrm{DTM}}_{\mathrm{new}}}$; low: < 0.1 m, medium: 0.1–0.3 m; high: > 0.3 m) of height values of the interpolated DTMs to assess the precision of each raster cell (Desmet, 1997; Chaplot et al., 2006; Fisher & Tate, 2006; Reuter et al., 2009; Wasklewicz et al., 2013).
2. We subdivided the terrain into raster cells with low point density (< 4 pts/raster cell), medium point density (4–10 pts/raster cell) and high point density (> 10 pts/raster cell). This classification was made based on our reference point cloud (Schöttlbach‐ALS). 4 pts/m^2^ was the minimum requirement and 10 pts/m^2^ the mean ground point density for the federal‐state‐wide flight campaign (Vermessung AVT‐ZT‐GmbH, 2015).
3. The whole study area was classified into flat to moderately steep areas (slope ≤ 30°) and steep terrain areas (slope > 30°).
4. Based on the formula of Karel and Kraus et al. (2006) the average height error (in centimetres) for a point cloud with 8 pts/m^2^ and 30° is about 31 cm. Since the vertical accuracy of Schöttlbach‐ALS, Lorenzerbach‐ALS1, Lorenzerbach‐ALS2, Rettenbach‐ALS1 with a mean ground point density of 8 pts/m^2^ is ±15 cm (Table 2; the models can therefore differ from each other by up to 30 cm) we decided to use 30° as class limit for slope.
5. Both point clouds were analysed by their point failures. Areas bigger than 6 m^2^ were considered as data gaps. These were further classified into gaps in flat or moderately steep (≤ 30°) and gaps in steep terrain (> 30°). This classification was based on the assumption that the influence of data gaps in flat or moderately steep areas on quality and uncertainties is negligible.
6. We used the DTM height accuracy map for both DTMs described in Section 3.4.1.

The information 1–5 for each raster cell served as input parameters for the final uncertainty analysis, which was done using a further development of the fuzzy inference system described by Wheaton et al. (2010). The aim of this analysis was to assign an uncertainty class to get the maximum height error for each raster cell. This threshold can be subsequently considered in the DoD and the geomorphic change detection (QCD‐tool).

**FIGURE 4:**
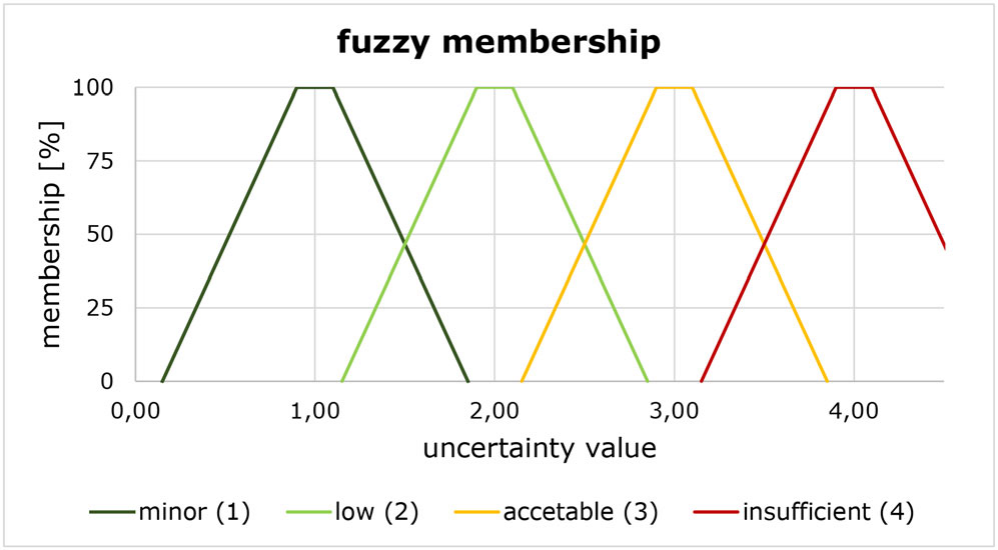
Fuzzy membership to the four uncertainty classes [Colour figure can be viewed at wileyonlinelibrary.com]

Each raster cell was assessed by analysing correlations of the individual input parameters RMSE, point density, slope, data gaps, and DTM height accuracy. Therefore 51 rulesets assigned to one of the four uncertainty classes were defined and provided with a class value (Table 3). Since each raster cell can be represented by several rulesets, a fuzzy membership to the four uncertainty classes (Wheaton et al., 2010; Zadeh, 1965) was defined (Figure 4, Table 3).

**TABLE 3:** Uncertainty analysis – four input fuzzy inference system for digital terrain model (DTM) uncertainty with 51 rulesets. The four inputs are root mean square error (RMSE), point density, DTM height accuracy and data gaps (slope) (n.c., not considered)

Ruleset	Inputs				Output	
	RMSE (m)	Point density (pts/cell)	DTM height accuracy (m)	Data gaps	Uncertainty	Class value
1	low	medium	low	n.c.	minor	1
2	low	high	low	n.c.	minor	1
3	low	medium	low	flat	minor	1
4	low	high	low	flat	minor	1
5	low	medium	low	steep	minor	1
6	low	high	low	steep	minor	1
7	medium	high	low	n.c.	minor	1
8	medium	medium	low	n.c.	minor	1
9	medium	medium	low	flat	minor	1
10	medium	high	low	flat	minor	1
11	medium	medium	low	steep	minor	1
12	medium	high	low	steep	minor	1
13	high	high	low	n.c.	minor	1
14	high	medium	low	n.c.	minor	1
15	high	medium	low	flat	minor	1
16	high	high	low	flat	minor	1
17	high	medium	low	steep	minor	1
18	high	high	low	steep	minor	1
19	low	medium	medium	n.c.	low	2
20	low	high	medium	n.c.	low	2
21	low	medium	medium	flat	low	2
22	low	high	medium	flat	low	2
23	low	medium	medium	steep	low	2
24	low	high	medium	steep	low	2
25	medium	high	medium	n.c.	low	2
26	medium	medium	medium	n.c.	low	2
27	medium	medium	medium	flat	low	2
28	medium	high	medium	flat	low	2
29	medium	medium	medium	steep	low	2
30	medium	high	medium	steep	low	2
31	high	high	medium	n.c.	low	2
32	high	medium	medium	n.c.0	low	2
33	high	medium	medium	flat	low	2
34	high	high	medium	flat	low	2
35	high	medium	medium	steep	low	2
36	high	high	medium	steep	low	2
37	medium	low	medium	n.c.	acceptable	3
38	medium	medium	good	n.c.	acceptable	3
39	medium	low	medium	flat	acceptable	3
40	medium	medium	good	flat	acceptable	3
41	medium	low	medium	steep	acceptable	3
42	medium	medium	good	steep	acceptable	3
43	low	low	good	n.c.	insufficient	4
44	medium	low	good	n.c.	insufficient	4
45	high	low	good	n.c.	insufficient	4
46	low	low	good	flat	insufficient	4
47	medium	low	good	flat	insufficient	4
48	high	low	good	flat	insufficient	4
49	low	low	good	steep	insufficient	4
50	medium	low	good	steep	insufficient	4
51	high	low	good	steep	insufficient	4

For each cell all class values were summarized and divided by the number of the applicable rulesets. Figure 4 shows the final assignment of the values to one of the four uncertainty classes by its percentage of membership.

This workflow allowed modelling of a certain degree of fuzziness and enabled a transparent assignment of each raster cell to one of the four uncertainty classes by defining its percentage of membership (Table 3).

## 4 RESULTS

The first step for a robust error and uncertainty analysis was to detect possible sources of error in the different datasets (cf. Section 3.4). The detailed evaluation of the uncertainty sources of the data used for this study is summarized in Table 4. Further accuracy analysis on the point clouds and DTM's (relative vertical accuracy, height residuals, percentage distribution of uncertainty classes by slope) was compiled in the Appendix.

**TABLE 4:** Summary of the evaluation of the main error sources of the two different point clouds of the Schöttlbach

Type	Description	Schöttlbach‐ALS	Schöttlbach‐ULS	Figure
*Data acquisition*				
Instrumental error	Artefacts respectively typical scan patterns in both point clouds			Figure 8
Methodical error	Different reasons for the flight campaigns	Large‐scale campaign	project‐based campaign	
	different flight heights	~ 130–1300 m above ground	~ 90 m above ground	
	different penetration of the vegetation due to the recording time and flight height	data were collected in summer during leaf‐on conditions	data were collected in late autumn during leaf‐off conditions	Figure 8
	shading effects on the side facing away from the sensor	‐	(1) lower and variable flight height (2) different scan angle (3) single, not overlapping flight strips	Figure 8
	different mean ground point densities	8 pts/m^2^	57 pts/m^2^	Figure 8
	different point spacing	regular planimetric point spacing	varying point spacing	Figure 8
	data gaps	13.2% of the catchment	7.6%	Figure 8
	different mean footprint	30cm	4 cm	
Random errors	Noise			
*Data processing*				
Errors in point clouds	Relative accuracy by stable surfaces over the area under investigation	Used as reference point cloud	Maximum deviation of the respective RMSE of the vertical position: 0.07 m	
	misclassification of ground and non‐ground points	good classification	manual post‐processing was needed	Figure 5
Errors in processed DTMs	Interpolated elevation values can partly deviate by less than 0.1 m from the original elevation values			
	simplification due to the raster resolution	DTM resolution: 50 cm	DTM resolution: 50 cm	
*Spatial properties of terrain*				
Surface errors	15% of the study area is steeper than 30°			
	meadows and coniferous forests (73%)			
	different level of detail of the geomorphology			Figures 5 and 8
	micro‐relief structures (especially in the riverbed)	are not mapped in the point cloud	are mapped in the point cloud	Figure 5
Physical errors	Signal attenuation and point failures at different surfaces (e.g., water, ice, etc.) caused by the used wavelength			Figure 8

**FIGURE 5:**
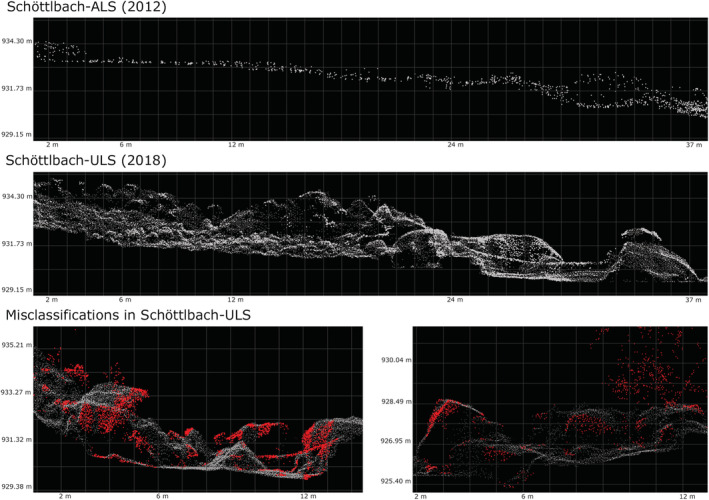
Level of detail of the representation of geomorphic structures in Schöttlbach‐ALS and Schöttlbach‐ULS caused by different laser footprints and point densities. Top: Cross‐section of an exemplary section of the ALS ground points, showing apparently smooth terrain. Middle: Same cross‐section represented in the ULS ground points, with a higher level of detail showing small boulders in the riverbed (left half of the section). Bottom: Misclassification in Schöttlbach‐ULS before manual re‐classification – red points are wrongly classified to non‐ground points in ULS data demonstrated on the basis of two different examples of the Schöttlbach‐ULS. [Colour figure can be viewed at wileyonlinelibrary.com]

According to our detailed evaluation, most of the errors detected in the data can be attributed to methodical errors (data acquisition). The different mean ground point densities together with different point spacings, footprints, flight heights, and the percentage of data gaps make the main differences between the compared LiDAR data and are therefore the main uncertainty sources of the data used in this study. The mean point densities of the different point clouds for example vary from 8 pts/m^2^ (Schöttlbach‐ALS) to 60 pts/m^2^ (Schöttlbach‐ULS). This is due to the different LiDAR sensors, flight heights and strip overlaps. The different point densities again result in a different level of detail of the geomorphic structures detected (Figure 5).

**FIGURE 6:**
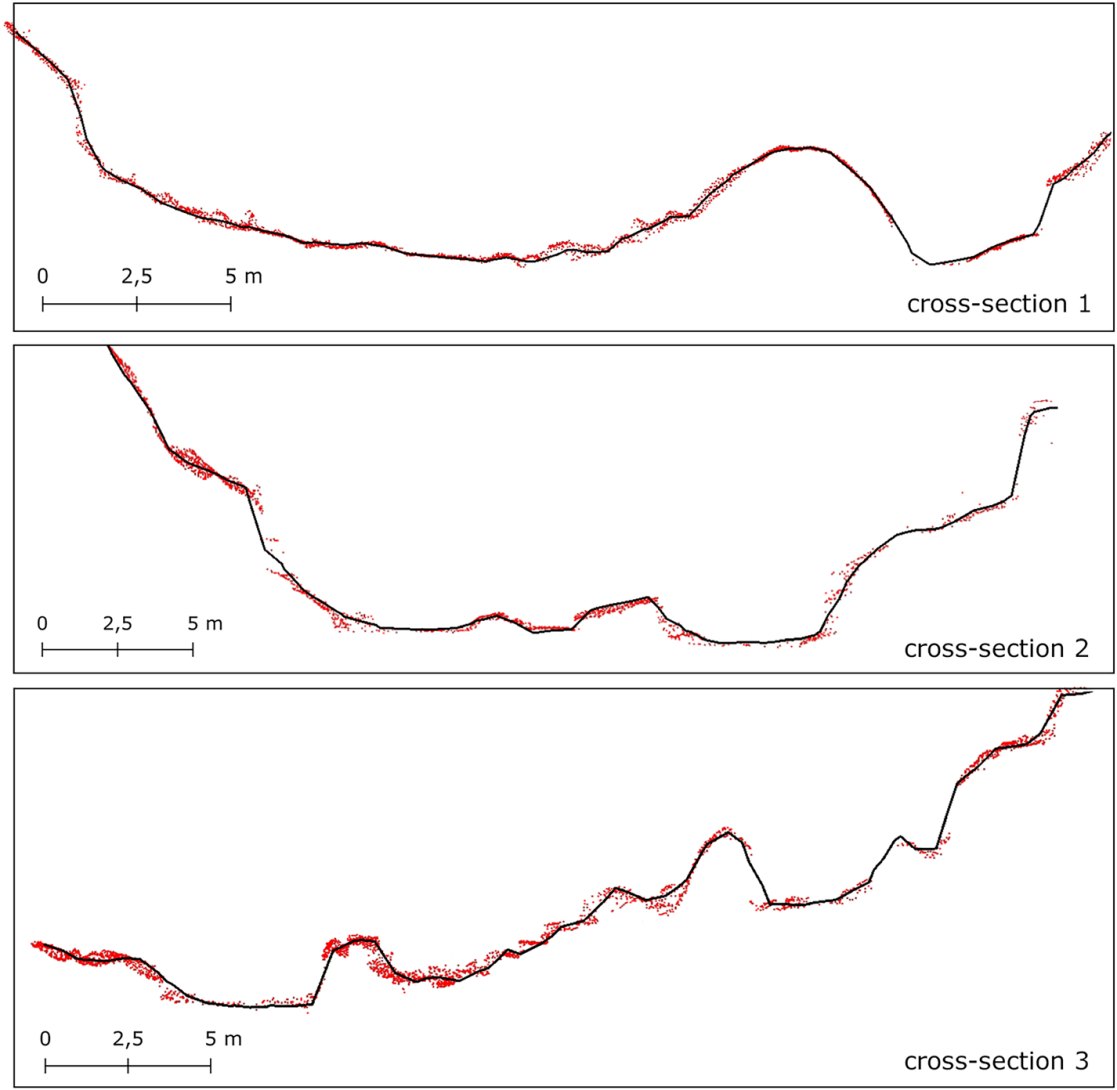
ULS point cloud and respective DTM with 50 cm resolution (2018; Schöttlbach‐ULS) illustrated in three cross‐sections of the lower part of the Schöttlbach creek (Figure 1) – point cloud surface representation (red points) versus DTM surface representation (black solid line). For a better visualization, all ULS ground points within a 50 cm buffer around the cross‐section are displayed. Thus, the high resolution of the Schöttlbach‐ULS and how much ULS points can scatter around the interpolated model are demonstrated. [Colour figure can be viewed at wileyonlinelibrary.com]

**FIGURE 7:**
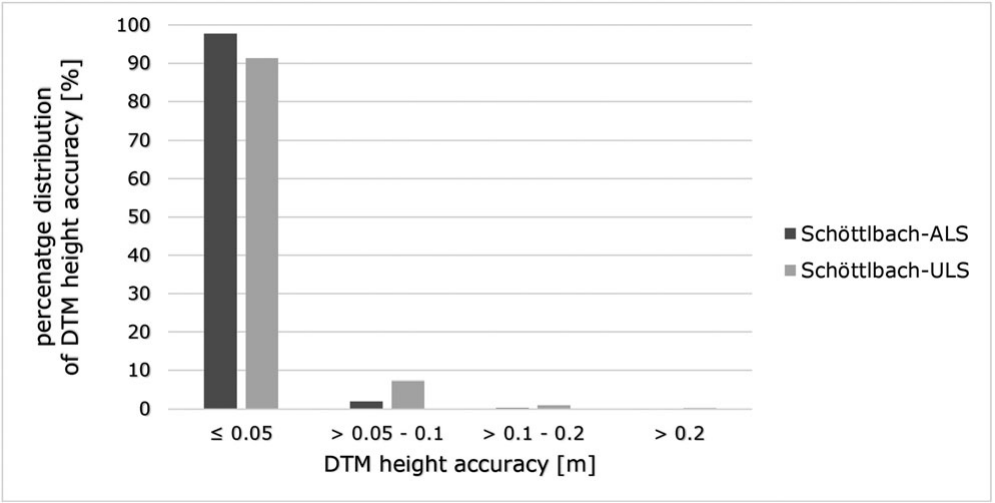
Percentage distribution of DTM height accuracy of the two point clouds in the Schöttlbach catchment. A DTM height accuracy of > 0.2 m in less than 0.5% in both point clouds.

With regard to errors in point clouds (data processing), a good relative accuracy (RMSE: 0.07 m) in both point clouds was found (Table 4; Section 4.2.). However, small inaccuracies (> 0.1 m; Figures 6 and 7) resulting from the DTM interpolation, and the raster resolution must be taken into account (Table 4, data processing). Concerning errors in processed DTMs, the original point cloud is simplified, and interpolated elevation values can partly deviate by less than 0.1 m from the original elevation values (Figure 5).

In addition, spatial properties of the terrain of the Schöttlbach like slope, different level of detail of geomorphic structures or radiation properties of different surfaces also have an influence on the vertical and horizontal accuracy and the quality of the final DTMs (Table 4, spatial properties of the terrain).

### 4.1 Uncertainties in multi‐temporal data

**FIGURE 8:**
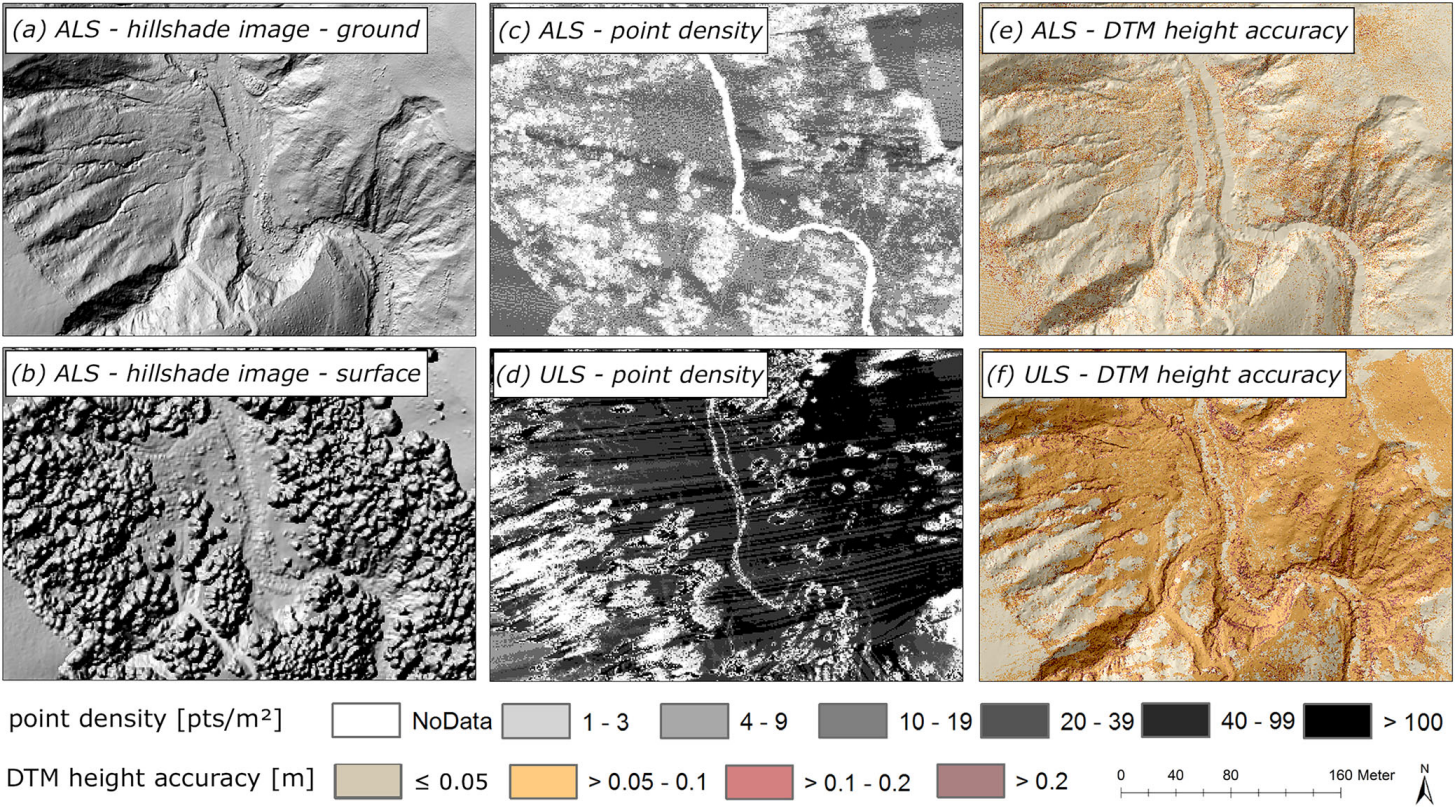
Evaluation of uncertainty sources of the Schöttlbach catchment: Hillshade of the DTM (a), hillshade of the DSM (b); point densities of the ALS (c) and the ULS point cloud (d); DTM height accuracy map visually showing a difference between the ALS point cloud (e) and the ULS point cloud (f). [Colour figure can be viewed at wileyonlinelibrary.com]

The result of the DTM height accuracy analysis (Section 3.4.1) shows a small difference in the accuracy/surface roughness between Schöttlbach‐ALS and Schöttlbach‐ULS (Figures 7 and 8e,f). DTM height accuracy of Schöttlbach‐ULS is slightly worse, with a higher portion in the range > 0.05 m. Nevertheless, the percentage distribution of DTM height accuracy is more than 90% for ranges lower than 0.05 m (Figure 7) and interpolated elevation values of both DTMs fit very well to the original point cloud (Figures 6 and 7).

Lower point densities are found under vegetated areas and in the area of the river channel in both point clouds (Figure 8b–d). On the side facing away from the scanner (UAV was navigated along the river) additional point failures can be seen in Schöttlbach‐ULS (Figure 8d). While a homogenous mean ground point density of about 10 pts/m^2^ with a planimetric point spacing can be found in Schöttlbach‐ALS (Figure 8c), Schöttlbach‐ULS has a much higher, but inhomogeneous mean ground point density of about 60 pts/m^2^ with a varying point spacing (Figure 8d). For more information concerning flight paths see Appendix (Figure A2).

**FIGURE 9:**
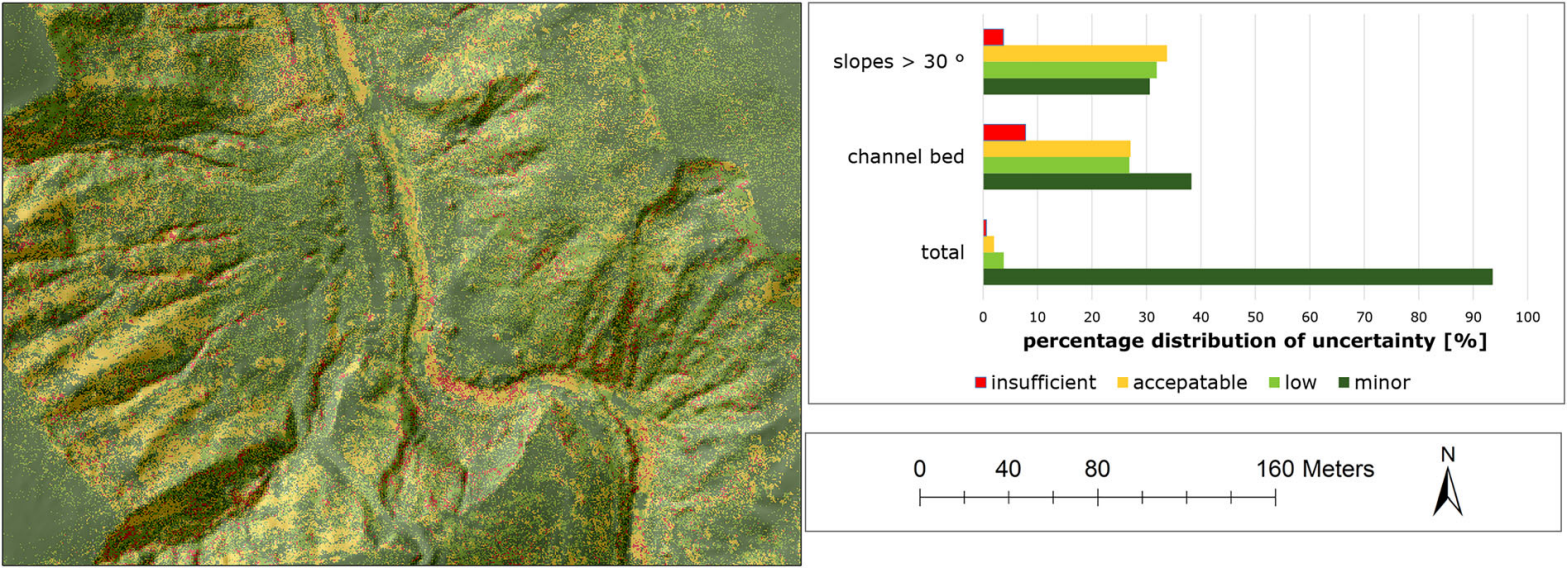
Uncertainty map (left) and percentage distribution of uncertainty classes (minor: 0.1/0.025; low: 02/0.05; acceptable: 0.3/0.075; insufficient: 0.4/0.1 [maximum height error [m]/volume uncertainty [m^3^/cell]]) in multi‐temporal DTMs of the Schöttlbach [Colour figure can be viewed at wileyonlinelibrary.com]

**FIGURE A3:**
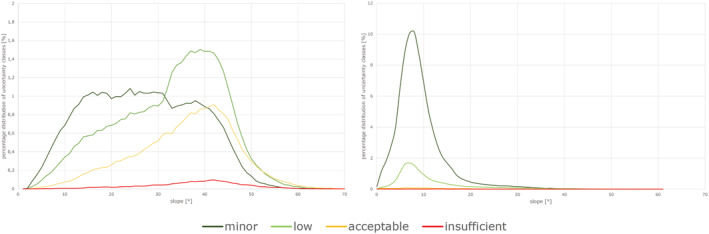
Percentage distribution of uncertainty classes by slope in the two evaluation areas (Lorenzerbach [left image]; Rettenbach [right image]). The biggest share of raster cells has a minor to low uncertainty. At Lorenzerbach a slope of > 30° lead to slightly higher uncertainties (low to acceptable). Rettenbach has a more gentle and flat terrain with raster cells with minor to low uncertainty. A good applicability of these multi‐temporal data for change detections in these torrential catchments is given. [Colour figure can be viewed at wileyonlinelibrary.com]

In general, the result of the uncertainty analysis (Section 3.4.2.) show that approximately 90% of all raster cells are assigned to minor uncertainties, which indicates a good comparability of the multi‐temporal DTMs (Figure 9). However, the riverbed, which in many studies is the main area of interest, is characterized by a slightly higher uncertainty and must therefore be considered more critically. In addition, slope areas (slope > 30°) are also characterized by higher uncertainties (Figure 9; Appendix Figure A3 and Table A2).

### 4.2 Evaluation areas

#### 4.2.1 Evaluation of uncertainty sources of evaluation areas

In the Lorenzerbach catchment, the difference of the mean ground point density between the older (8 pts/m^2^) and the more recent survey from 2012 (13 pts/m^2^) is relatively small. At the Rettenbach catchment, the second ALS point cloud from 2018 has a higher point density (27 pts/m^2^) than the first one from 2009 (8 pts/m^2^) and points are more evenly distributed over the whole study area.

All ALS datasets show a regular planimetric point spacing over the whole flight area that can be also seen in the point density maps as well as the DTM height accuracy maps. The three point clouds of the federal‐state‐wide flight campaign are similar concerning a lack of ground points in the LiDAR point clouds (Lorenzerbach‐ALS1, Lorenzerbach‐ALS2 and Rettenbach‐ALS1: between 12.0–15.0%). Concerning percentage of data gaps, Rettenbach‐ALS2 has the lowest share (2.8%).

#### 4.2.2 Uncertainties in multi‐temporal data of evaluation areas

**FIGURE 10:**
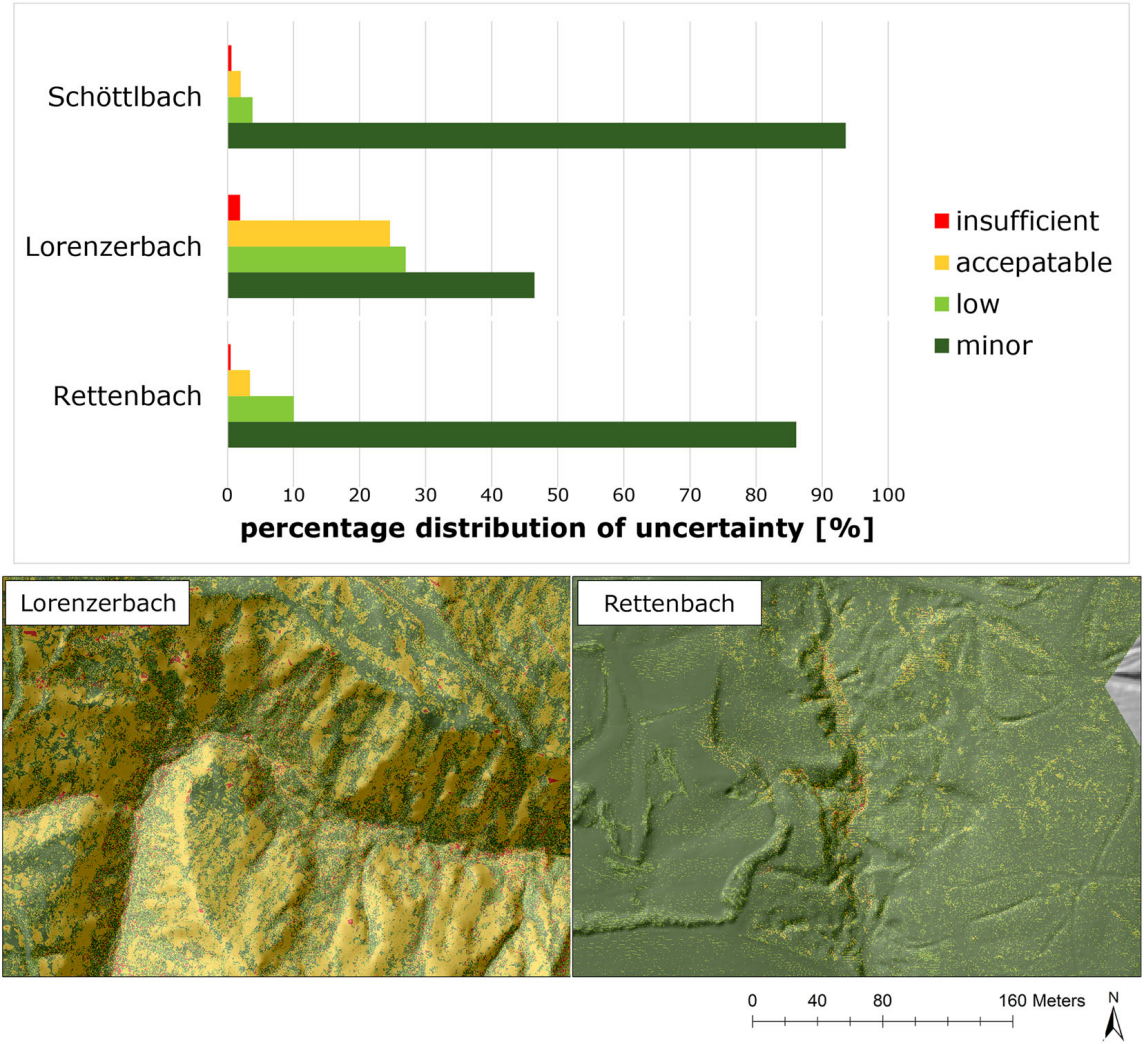
Comparison of the percentage distribution of uncertainty classes in multi‐temporal DTMs of Schöttlbach (cf. Figure 9), Lorenzerbach and Rettenbach (top) and uncertainty maps of Lorenzerbach and Rettenbach (bottom) [Colour figure can be viewed at wileyonlinelibrary.com]

DTM height accuracy maps show a difference between the reference point clouds and the more recent point clouds in both evaluation areas. Interpolated elevation values of Lorenzerbach – especially the second survey – show higher deviations (> 0.1–0.2 m). The interpolated elevation values of Rettenbach fit very well to the original point cloud (maximum deviations of less than 0.1 m). The results of the uncertainty analysis show minor to low uncertainties in the single raster cells (Lorenzerbach: 60%, Rettenbach: 90%) (Figure 10).

### 4.3 Applicability of the uncertainty analysis for geomorphic impact studies and volumetric change

For volumetric change calculations, a maximum height error for each raster cell and each uncertainty class was defined (Table 5). The maximum height error for raster cells with minor uncertainty is based on the DTM height accuracy analysis described in Section 3.4, step 1. This was done for each study/evaluation area individually, which is the reason for the differences in maximum height error between Schöttlbach and Rettenbach (0.10 m) and Lorenzerbach (0.20 m). For example, within the Schöttlbach catchment, class 1 means a maximum height error of 0.1 m and a volume‐uncertainty of 0.025 m^3^/cell, while class 4 means a maximum height error of 0.4 m and a volume uncertainty of 0.1 m^3^/cell.

**TABLE 5:** Maximum height error (m) and volume uncertainty (m^3^/cell) of each raster cell for each uncertainty class

Uncertainty class	Schöttlbach and Rettenbach (raster resolution: 0.5 m)		Lorenzerbach (raster resolution: 1.0 m)	
	Maximum height error (m)	Volume uncertainty (m^3^/cell)	Maximum height error (m)	Volume uncertainty (m^3^/cell)
Minor	0.1	0.025	0.2	0.2
Low	0.2	0.05	0.3	0.3
Acceptable	0.3	0.075	0.4	0.4
Insufficient	0.4	0.1	0.5	0.5

According to the WLV, a sediment volume of 100,000 m^3^ (no uncertainty range provided) was transported from the catchment area Schöttlbach in August 2017. We calculated a volume of 131,000 m^3^ (± 61,000 m^3^) of transported sediments using the QCD‐tool (detailed information are available in Krenn et al., in review).

In comparison, a volume of about 123,000 m^3^ of sediments were mobilized in the catchment area Lorenzerbach in July 2012 due to WLV estimates (Janu & Mehlhorn, 2013) and about 300 m^3^ in the Rettenbach area in May 2013 (no uncertainty range provided for both areas). These amounts are relatively close to the results of our change detection analysis (Lorenzerbach 90,000 m^3^ ± 35,000 m^3^; Rettenbach 800 m^3^ ± 450 m^3^; based on the QCD‐tool described in Krenn et al., in review).

**FIGURE 11:**
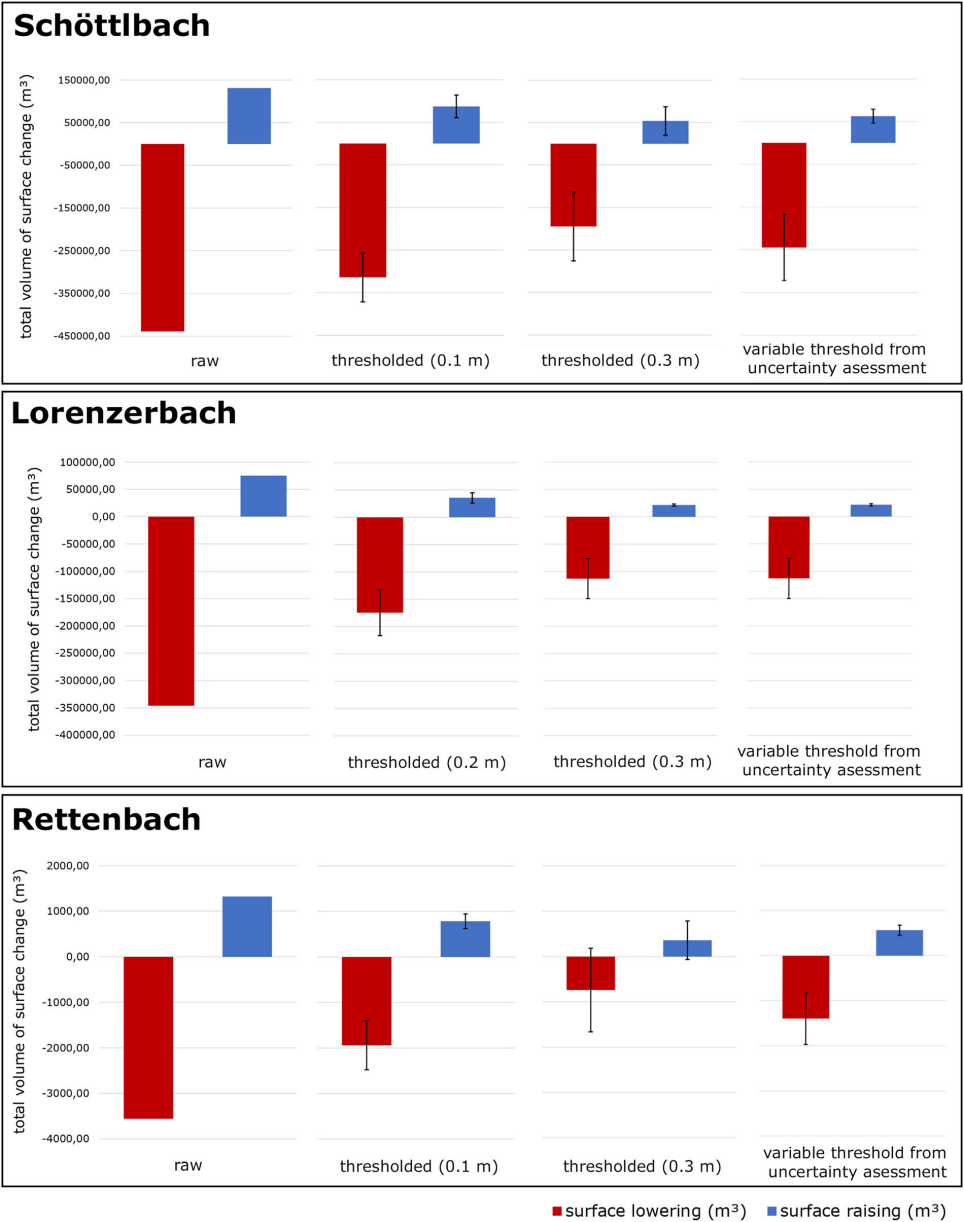
Results of the change detection analysis (volumetric changes [m^3^]) of the three catchments Schöttlbach, Lorenzerbach and Rettenbach [Colour figure can be viewed at wileyonlinelibrary.com]

Figure 11 displays how calculated volume changes vary by integrating the newly developed uncertainty assessment in the QCD‐tool. The unfiltered change detection delivers unrealistically high values. Using a fixed threshold of 0.1 or 0.3 m height change gives very different results which are highly dependent of the value of the threshold applied. The values of our QCD‐tool with a variable threshold depending on uncertainty classes lie between the results of the fixed thresholds of 0.1 and 0.3 m (approximately half as high as the raw difference model).

## 5 DISCUSSION – MAGNITUDE OF ERRORS AND UNCERTAINTIES

### 5.1 Error sources and uncertainties in data acquisition

A central point of our work and in the development of our workflow was the use of already existing data (e.g. data financed by the public sector) instead of commissioning a new flight campaign. In this case there is little to no influence on used LiDAR sensors and errors caused by data acquisition.

When analysing these errors in LiDAR data, parameters like the phenological status (leaf‐on or leaf‐off), the wetness of surfaces, flight height and perspectives (resolution and nadir vs. oblique) (Mandlburger et al., 2015a), the purpose of the survey (project‐based or large‐scale campaign) or the used LiDAR sensor must be considered.

Compared with the Schöttlbach‐ALS, Schöttlbach‐ULS shows high point density and significantly less data gaps due to the high scan rate. However, due to the lower and variable flight height, and the different scan angle of the UAV, shading effects were caused on the side facing away from the sensor even though the data were collected in late autumn during leaf‐off conditions (Figure 8d) (Zhou et al., 2020). This results in a slightly irregular planimetric point spacing over the whole area. In principle, this problem could be avoided by adding additional flight trajectories (Brede et al., 2017; Mandlburger et al., 2015a).

Rettenbach‐ALS2 was also acquired during leaf‐off conditions. The ALS flight strips were not only acquired with a high overlap of 50% and a strip spacing of 500 m between the parallel strips, but data were also collected by using a cross‐flight strategy. When measuring a smoother terrain such as the Rettenbach catchment, a higher point density is achieved showing a regular planimetric point spacing and less data gaps. This resulted in a high‐quality dataset with few data gaps and objects and structures acquired from various directions.

### 5.2 Error sources and uncertainties in data processing

The results of the accuracy analysis show a high relative vertical and horizontal accuracy of the point clouds (RMSE – Schöttlbach: 0.07 m; Lorenzerbach: 0.10 m; Rettenbach: 0.07 m; Table A1). The slightly lower accuracy of Lorenzerbach‐ALS2 is attributable to the boundary of the surveyed area being located along the high alpine ridges of the catchment area. In this remote terrain, less stable surfaces can be used/found for quality purposes, leading to lower accuracies (see also Figures A1a and A1b). Therefore, an increase of the area to be surveyed to include areas/surfaces suitable for georeferencing would improve accuracies.

Despite the increase in available high‐density ground points and resulting from this also of the DTM accuracy (Bakuła et al., 2020; Kraus et al., 2006), a DTM still remains a modelled representation of the surface and does not fully represent the exact landforms, geomorphic features, and hence surface processes (Hengl & Reuter, 2009). One problem is that a raster data model is based on a regular grid and raster points are not necessarily located along terrain breaklines. In our analysis, the latter, together with the high point densities of the ULS point cloud (Figure 5), are the main reasons why interpolated elevation values can partly deviate from the original elevation values (Figures 5 and 7).

We used DTMs instead of point clouds for change detection due to different point densities of the datasets. Therefore, larger uncertainties caused by DTM interpolation were accepted for a good comparability of the multi‐temporal data (Figure 10).

The high point densities of ULS point clouds is not only an advantage, but can also cause problems, for example misclassifications of small‐scale height differences of rocks and boulders as non‐ground points (Figure 5) (Zhou et al., 2020). Kraus and Pfeifer (2001) also found that the combination of very dense datasets and areas with low vegetation and high surface roughness can lead to misclassifications of ground and non‐ground (vegetation) points.

If used for DTM interpolation, these falsely classified ground points would cause errors in volume quantifications.

Therefore, a manual re‐classification of the ULS ground points focusing on the small‐scale structures along and in the riverbed is required. Misclassified boulders (classified as vegetation) in the river channels would affect any kind of hydro‐morphological analysis. Otherwise, data gaps, differently treated by various interpolation algorithms, might lead to a biased terrain representation. For this reason, the interpolation algorithm has to support the research question and the quality of the original point cloud. In other words, the uncertainty of data must be of smaller magnitudes then the magnitudes of changes caused by geomorphic processes. For a reasonable estimation of geomorphic impacts, we recommend point cloud filtering and classification adapted to (and/or designed for) the research purpose and the point clouds of different origins and/or epochs. Therefore, if that is possible, the raw point cloud should be used instead of the final DTM. We found that meaningful results are much easier to obtain when all DTMs are interpolated with the same algorithm, tools, or the same software product to create DoDs that are suitable for the research question. Using multi‐temporal data generated with different algorithms and workflows may cause additional uncertainties.

### 5.3 Error sources and uncertainties in spatial properties of terrain

Steep slopes, high surface roughness and in parts densely forested areas lead to errors and uncertainties in our data (e.g., comparably coarse mean ground point density; Figures 5 and 8c,d). The result of the DTM height accuracy analysis shows a clear difference in the height accuracy (surface roughness) between Schöttlbach‐ALS and the Schöttlbach‐ULS. This is caused by a higher level of detail due to higher point densities and a lower flight height of the ULS (Figure 5).

Since all used sensors are operating in the NIR range (about 800 to 2500 nm), signal attenuation and point failures are forced by water bodies and in the water‐filled river channel (therefore, greenlight scanners could possibly lead to better results). That means that morphology of the riverbed must be treated and interpreted carefully (Figures 5 and 6).

Different flight heights result in different scanning angles, level of detail of the point clouds and point densities. These differences affect the level of detail in terrain structures, objects, and edges. The footprint (Schöttlbach‐ALS: 30cm; Schöttlbach‐ULS: 4 cm) of the laser beam, which is influenced by the laser beam divergence and the flight height, also limits the accuracy of the data and the size of objects and structures that can be detected (Figure 5). Subsequently, small structures like boulders in the river channel are not recorded and slight movements of small boulders cannot be detected (Pfeifer & Briese, 2007). Due to the larger flight height of ALS surveys, steep slopes are much better represented in the ULS dataset.

The study area and evaluation areas vary in levels of terrain roughness because of different geological properties, land‐cover and land‐use properties (Figures 1 and 2). A smooth and anthropogenically homogenized terrain with meadows and deciduous/mixed forests (> 70%) are the main reasons for lower surface roughness levels in the Rettenbach catchment. A slightly higher surface roughness in the Schöttlbach area is caused by a higher percentage of coniferous forests (> 50%) and steep slopes (> 40%). Whereas, in the Lorenzerbach catchment, coniferous forests and steep slopes are predominant. These two characteristics are causing a rough terrain in the Lorenzerbach catchment. This is also reflected in the results of the DTM height accuracy and uncertainty analysis (Figures 7, 8, 9 and 10). Therefore, higher maximum height errors for each uncertainty class were accepted for the Lorenzerbach catchment (Table 5). Even if for example Zhou et al. (2020) achieved good results in their UAV‐based investigation of archaeological features under dense forest, however in a more subdued topographical setting, as a general result we would recommend that in steep, forested areas individual plausibility checks are generally advisable to distinguish between real geomorphic changes and apparent changes due to different errors.

### 5.4 Error sources and uncertainties in multi‐temporal data

In the analysis of the results of the uncertainty workflow, all presented data show sufficient quality for our geomorphic impact study to detect surface changes of more than ±0.5 m. The approach based on the work of Wheaton et al. (2010) turned out to be very promising in terms of calculating sediment budgets, even if it was not specifically designed for high‐resolution LiDAR point clouds. Hence, we adapted and refined their work by taking into account the high point density of the used data in our workflow. Wheaton et al. (2010) estimate error rates in a DTM using a probabilistic approach with a fuzzy inference system (FIS), where information about the point quality, point density and slope is taken into account (Mandlburger et al., 2015b). The bootstrapping approach applied by Wheaton et al. (2010) was not used in our workflow for following reasons: removing a random sample of 10% of the points however does not really have a significant impact on an interpolated DTM of 50 cm spatial resolution with point densities as we can find within ULS data. In contrast to point clouds with low point densities, terrain breaklines might be filtered out and decrease the quality of the DTM. Without preserving terrain breaklines, thinning out point clouds in a rugged terrain (e.g., in a torrential catchment), is therefore not recommended. Instead of applying the bootstrapping approach, we compared the interpolated elevation values with original elevation values of the raw point cloud to calculate height deviations (roughness). The raw point cloud was defined as true representation of the terrain (similar as in Kraus et al. [2006]).

Our DTM height accuracy analysis provides important insights about how well interpolated elevation values match to the original point cloud (original surface) and hence supports the following geomorphological analysis. In principle, the higher the point density and spatial coherence of the point clouds, the more accurate the surface roughness is represented (Mandlburger et al., 2015b), which can be seen in Figure 7 (Schöttlbach‐ULS). Small deviations based on the internal accuracy and precision of the sensor systems (Riegl LMS‐Q560/LMS‐Q680i: 20/20 cm; Riegl VUX‐1LR: 15/10 cm; Riegl VQ‐780i: 20/20 cm) and inaccuracies in the strip adjustment are also reflected in the final DTM height accuracy map (Figures 7 and 8). Our DTM height accuracy analysis (see Section 3.4.1) can also be used for other modelling purposes like simulating runoff or bedload, floods, rockfall or natural hazard modelling (Brožová et al., 2021; Haas et al., 2012; Zhang et al., 2021).

### 5.5 Geomorphic impact studies and volumetric change

Results of our geomorphological change detection approach underline the important impact of a detailed uncertainty analysis. By using the raw change detection, volume changes are highly overestimated. Thresholding the studied area with a mean height error would save a lot of extra work, but degrades estimates of net change by missing/removing some real geomorphic change (Figure 11) (Anderson, 2019). In our study areas the differences between thresholding versus quantifying uncertainty in change detection is substantial. A detailed analysis of error propagation and the role of thresholding in topographic change was done by Anderson (2019).

For comparing sediment relocation estimated by local experts with the results of our change detection approach, some fundamental error sources have to be kept in mind. Estimates, in our case by the WLV, are no more and no less than educated guess. At Lorenzerbach, the difference between estimate (123,000 m^3^) and change detection (90,000 m^3^ ± 35,000 m^3^) might be partly due to the lower density of freshly redeposited material compared to consolidated slope sediments, or to transport out of the investigated catchment area. More detailed information of the volumetric change assessment at Schöttlbach are provided in Krenn et al. (in review).

## 6 CONCLUSIONS – COMPARABILITY OF MULTI‐TEMPORAL LIDAR DTMS

Even though the technical setup of airborne and UAV‐borne LiDAR is similar, there are differences between the different sensor systems (Table 2) and major differences in the characteristics of the differently acquired point clouds, the interpolated DTMs, and their inherent uncertainties. We developed a workflow for DTM generation and geomorphic change detection with the rigorous consideration of uncertainty analysis and applied it to three catchments. The presented results show that the combination of ULS and ALS data (or of ALS datasets of different quality), aided by our uncertainty workflow, are promising for calculating volumetric changes at catchment‐scale in a decimeter resolution (> ±0.5 m).

In addition to a data acquisition adapted to the research question, point clouds acquired with new LiDAR sensors could reduce uncertainties and improve geomorphic change detection results. This could be investigated in more detail in a next step.

Our findings reveal that the high point density of ULS data is very well suited for analysing the surface roughness, making ULS data particularly useful for forest, vegetation, or micro‐scale applications (e.g., identification of micro‐scale landforms, detailed river morphology, etc.). However, the low flight height of UAVs leads to shadowing effects and subsequently low point densities or even data gaps in the point clouds. This leads to an incomplete representation of objects (Figure 8d). This problem can be solved by a different flight planning: instead of acquiring data with one single strip along a river channel, a cross‐flight would be preferable.

Several uncertainties and challenges arise from comparing ALS and ULS datasets, exemplified by our case study in the catchment area Schöttlbach.

Our workflow shows one option how to handle these high‐resolution and different LiDAR data. We recommend that the original point clouds serve as basis for the workflow. Methodical errors in data acquisition can be reduced by informing the contracted companies about desired flight planning. If this cannot be influenced, a detailed post‐processing chain can improve results by re‐filtering or reclassifying the point clouds, by improving the height adjustment and by interpolating DTMs with the workflows and algorithms as similar as possible and adapted to the scientific question. Nevertheless, data processing can either reduce different errors but also cause further problems such as a deterioration of an absolute and relative accuracy or a blurring of terrain breaklines. A detailed evaluation of the original point clouds by visual but also statistical approaches (data acquisition, data processing, spatial properties of the terrain) provide helpful insights into the comparability of the used data.

The high point density of the ULS point cloud does not necessarily improve the analysis of the geomorphic impact study, because the detailed information in a ULS point cloud (e.g., micro‐relief, land‐cover type) makes classification of the point clouds and geomorphic interpretation challenging and makes it harder to compare the surface models with those from lower‐resolution ALS data. Therefore, it is questionable whether the high level of detail in ULS datasets really adds value to geomorphic impact studies at catchment scale, as the full potential of these data can only be exploited when being compared to a dataset with similar accuracy and quality. This could also be a high‐resolution, preferably cross‐flight ALS dataset, as underlined by the satisfactory results of Rettenbach‐ALS2.

In our change detection analysis for the three catchments, we calculated sediment relocation of 130,000 m^3^, 90,000 m^3^ and 800 m^3^, respectively, using our novel uncertainty analysis workflow. All values are well in the magnitude of previous expert estimates. Using raw data or relatively low change detection thresholds (e.g., 0.1–0.2 m) results in a considerable over‐estimation of sediment volumes, while a rigid higher threshold (e.g., 0.5 m) results in presumably too low volumes, as real surface changes in areas with good data quality are unnecessarily omitted. Our dynamic threshold based on uncertainty calculations could therefore be an improvement on the approach of Wheaton et al. (2010).

## CONFLICTS OF INTEREST

The authors declare that there are no conflicts of interest that could be perceived as prejudicing the impartiality of the research reported.

## Figures and Tables

**FIGURE A1a:**
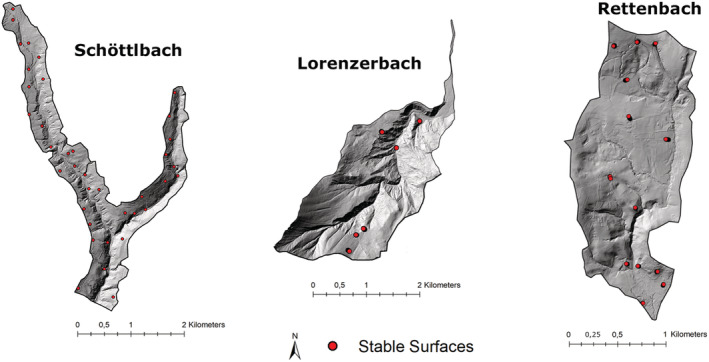
Stable surfaces of the study area and evaluation areas. Due to its distinctive topography only a few GCPs could be found in the Lorenzerbach catchment. [Colour figure can be viewed at wileyonlinelibrary.com]

**FIGURE A1b:**
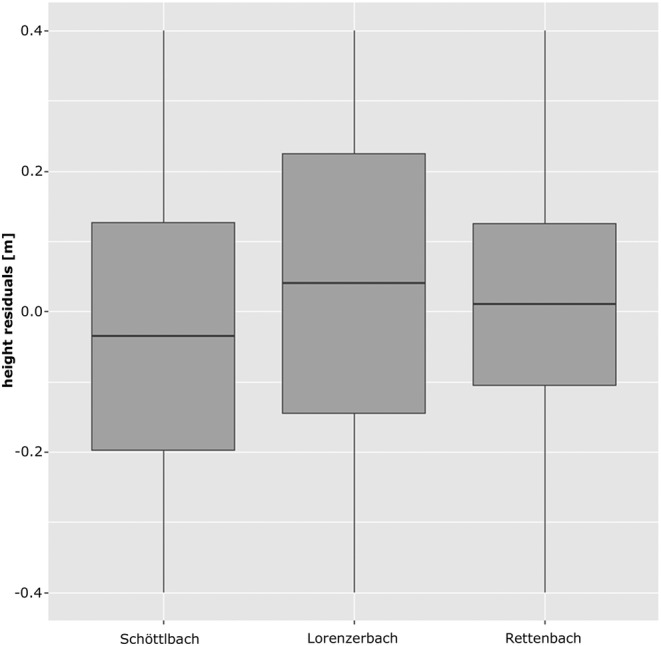
Boxplots of height residuals of GCPs. Absolute elevation values of point clouds deviate by only a few centimetres from the older point clouds (Schöttlbach‐ALS, Lorenzerbach‐ALS2, Rettenbach‐ALS2). Based on this evaluation, a value of 0.1 m (maximum deviation of the respective RMSE of the vertical position of the single study areas: Schöttlbach: 0.07 m; Lorenzerbach: 0.10 m; Rettenbach: 0.07 m) was used as mean height error in the final uncertainty models. The boxplots of the height residuals of the different point clouds shows that elevation values of the more recent point cloud of Schöttlbach are on average slightly lower (~0.04 m) than Schöttlbach‐ALS, whereas elevation values of Lorenzerbach‐ALS2 (~0.03 m) and Rettenbach‐ALS2 (~0.01 m) are slightly higher.

## Data Availability

The raw data used in this study are available from third parties (GIS Steiermark, Stadtvermessungsamt Graz and WLV). The availability of these data, which were used under license for this study, is subject to restrictions.

## APPENDIX A

### A.1 Relative vertical accuracy of LiDAR data

In preparation for analysing uncertainties in LiDAR data, stable surfaces (GCPs) which can be detected in both point clouds with different orientations evenly distributed over the area under investigation were selected and evaluated. This was done to verify the relative vertical and horizontal accuracy of the different point clouds (Figure A1).

The results verify a good height adjustment (Table A1). Based on this evaluation, a value of 0.1 m (based on the RMSE) is used as minimal threshold in the final uncertainty models.

**TABLE A1:** Relative vertical accuracy (m) of the study area and the two evaluation areas. Parameters like root mean square error (RMSE), mean error (ME), standard deviation (SD) or mean absolute error (MAE) are used to assess the precision of a dataset (Desmet, 1997; Chaplot et al., 2006; Fisher & Tate, 2006; Reuter et al., 2009, 2009; Wasklewicz et al., 2013). The RMSE is the most common metric used to characterize vertical errors and represents a direct comparison between measured or calculated ( Z) to reference height values ( $Z_{\mathrm{ref}})$ for a sample of *n* points (Reuter et al., 2009)

	Relative vertical accuracy (m)		
	Schöttlbach	Lorenzerbach	Rettenbach
MAE	0.05	0.07	0.06
RMSE	0.07	0.10	0.07
SD	0.06	0.08	0.04
ME	0.04	0.07	0.06

### A.2 Flight paths of the different LiDAR point clouds

Specialties: Schöttlbach‐ULS: Due to the elongated shape of the study area the flight plan for the ULS campaign followed two longitudinal lines along the river course; Lorenzerbach‐ALS1: This point cloud is merged from two flight campaigns from 2 years (2009/2011); Rettenbach‐ALS2: This point cloud is the result of a cross‐flight strategy.

### A.3 The influence of slope and the water‐filled channel bed on point density and uncertainty

As a supplement to Section 4, the influence of slope and the water‐filled channel bed on mean ground point density and uncertainty were also considered in more detail (Table A2 and Figure A3).

**TABLE A2:** Mean ground point density (pts/m^2^) differentiated by slope > 30° and channel bed. This verifies a sufficiently good mean ground point density in these areas. The results of Schöttl‐ULS underline that the flight‐campaign was project‐based designed to study the channel bed of the Schöttlbach

Mean ground point density (pts/m^2^)						
	Schöttl‐ALS	Schöttl‐ULS	Lorenzer‐ALS1	Lorenzer‐ALS2	Retten‐ALS1	Retten‐ALS2
Whole area	8.0	57.0	8.0	13.0	8.0	27.0
Slopes > 30 °	7.0	55.0	7.0	12.0	6.0	22.0
Channel bed	8.0	66.0	8.0	13.0	6.0	23.0
